# A study on the response of FRET based DNA aptasensors in intracellular environment

**DOI:** 10.1038/s41598-020-70261-1

**Published:** 2020-08-06

**Authors:** Shreya Ghosh, Yinghua Chen, Jesvin Sebastian, Anne George, Mitra Dutta, Michael A. Stroscio

**Affiliations:** 1 Department of Bioengineering, University of Illinois at Chicago, 851 South Morgan Street (SEO 218), Chicago, IL 60607 USA; 2 Department of Oral Biology, University of Illinois at Chicago, 801 South Paulina Street, Chicago, IL 60612 USA; 3 Department of Electrical and Computer Engineering, University of Illinois at Chicago, 851 South Morgan street, M/C 154, Chicago, IL 60607 USA; 4 Department of Physics, University of Illinois at Chicago, 845 W. Taylor St., M/C 273, Chicago, IL 60607 USA

**Keywords:** Oligonucleotide probes, Biosensors

## Abstract

This paper presents a study of the response of FRET based DNA aptasensors in the intracellular environment. Herein, we extend previous studies of aptasensors functioning in the extracellular environment to detection of antigens in the intracellular environment. An essential step in this research is the use of a novel means of achieving the endocytosis of aptasensors. Specifically, it is demonstrated that functioning aptasensors are successfully endocytosed by functionalizing the aptasensors with endocytosis—inducing DSS peptides.

## Introduction

Biomarker proteins are an important indicator of the presence of a specific disease condition in the physiological system. Hence, sensors for biomarker protein detection are being widely studied today. Techniques like Enzyme Linked Immunosorbent Assays (ELISAs) have been traditionally used to determine biomarker proteins like Tumor Necrosis Factor-alpha (TNF-α). TNF-α has been determined to be an important biomarker for infectious conditions like sepsis^1^ while Glycated albumin (GA) has been observed to be a more versatile biomarker of diabetes mellitus for all kinds of patients including the ones with blood based disorders^2^. Therefore, the objective of this study is to detect these biomarkers in an intracellular environment in order to facilitate early detection of these diseases; this is possible due to the pioneering work on the use of DSS as an endocytosis-inducing peptide. Ghosh et.al have summarized previously published detection techniques for TNF-α and GA and compared the advantages of their FRET based DNA aptasensors with those of the published sensing platforms^1,2^.

DNA aptamers are short oligonucleotides, which have the capability of binding to another molecule. Aptamer based sensors have been evaluated to have excellent recognition capacity towards various types of target molecules. These antigens include metal ions like lead^3^, mercury^4^, potassium^5–7^ as well as biomolecules like thrombin^8^, interferon-γ^9^, ATP^10^, AMP^11^ etc.

Fluorescence resonance energy transfer (FRET) is a process, which facilitates transfer of energy from a ‘donor’ nanostructure to an ‘acceptor’ nanostructure. The efficiency of the FRET process is proportional to 1/{1 + (d/d_o_)^6^} where d is the distance between the donor and the acceptor and d_o_ is generally approximately 5 nm^12–14^. The 1/{1 + (d/d_o_)^6^} dependence results from a dipole–dipole interactions between the donor and the acceptor. For a typical case, the FRET effect is relatively strong when d is less than about 5 nm and is relatively weak when d is greater than about 5 nm. In this study, the donor is a semiconductor quantum dot (QD) and the acceptor is a gold (Au) nanoparticle. When the QD and the Au nanoparticle are closer than about 5 nm there is strong transfer of energy from the QD to the Au nanoparticle and there is consequently less energy available in the quantum dot to emit as photons. Accordingly, when the QD and the Au nanoparticle are closer together the light emitted by the QD decreases significantly. The quantum dot donor and a gold nanoparticle acceptor are bound to opposite ends of a DNA aptamer. The DNA aptamer acts as the primary sensing element since it changes its conformational shape—and therefore its length—when the DNA aptamer binds selectively to the analyte. The change in the conformational shape of the aptamer causes a change in d and, therefore, causes a change in the intensity of the light emitted by the QD. It is this change in the intensity of the light emitted by the QD that indicates that the QD and the Au nanoparticle have changed their separation, d, as a result of the conformation-changing binding event between the aptamer and the analyte. Hence, the presence of the analyte is detected based the change in the intensity of the light emitted by the QD. Such a QD-aptamer—Au-nanoparticle complex is an example of a molecular beacon, in this study, it is the FRET-based molecular beacons that are used to detect the presence of the analyte.

## Results and discussion

### Characterization of DSS peptide structure

**Figure 1:**
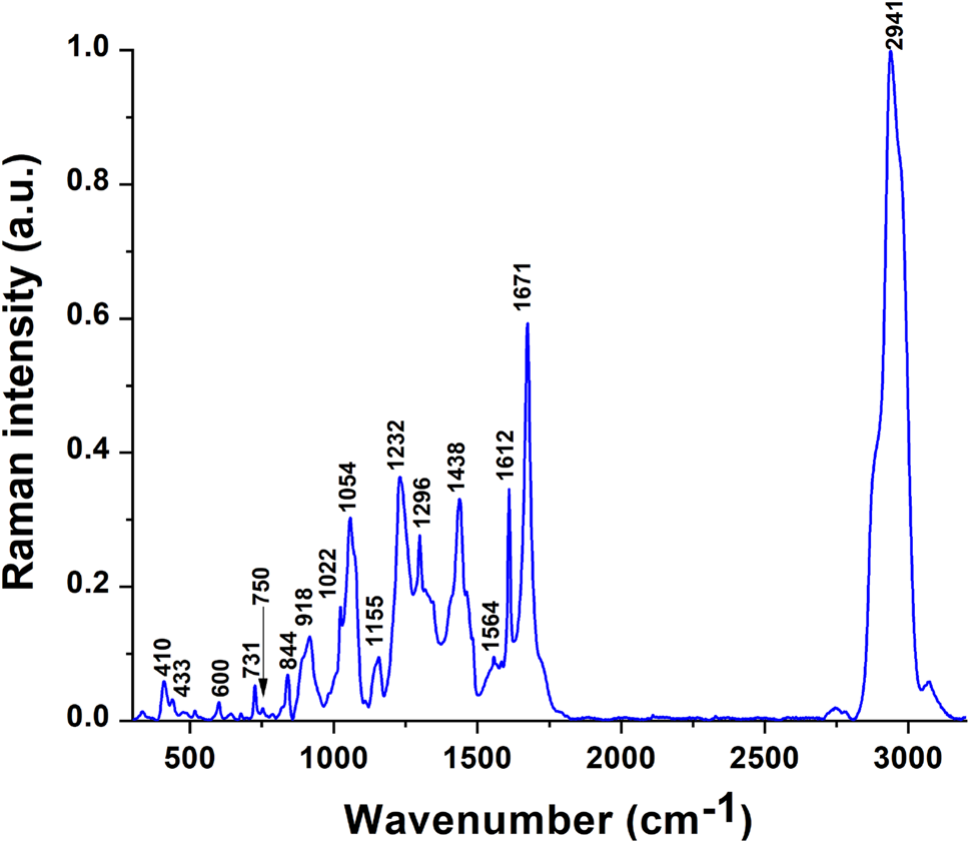
Raman spectra of the DSS peptide measured from 300 to 3,100 cm^−1^.

Figure 1 represents the Raman spectra of the DSS peptide from 300 to 3,100 cm^−1^ while Supplementary Table S1 summarizes the peak assignments for the spectra obtained for the peptide. In Fig. 1, there are three peaks (410 cm^−1^, 433 cm^−1^ and 600 cm^−1^), which were unidentified and hence does not have any possible characteristics. The peak at 1,232 cm^−1^ corresponds to the Amide 3 region of the peptide while the peak at 1564 cm^−1^ falls in the Amide 2 region of the peptide^15^. Additionally, the peak at 1564 cm^−1^ might also be contributed by COO^−^ stretching in aspartic acid^16^. The peak at 1671 cm^−1^, which corresponds to the Amide 1 region of the peptide, also indicates the presence of antiparallel beta sheet structure^15^. Previous studies have shown that the beta sheet structure is important for cellular uptake^17–19^. Therefore, the structural information from the Raman spectra provides insight into the cell penetrating attribute of the peptide. The peak characteristics stated in Supplementary Table S1 have been obtained from previously published literature^15,16,20–25^.

### Testing DSS peptide conjugated molecular beacons with PBS based TNF-α samples

**Figure 2:**
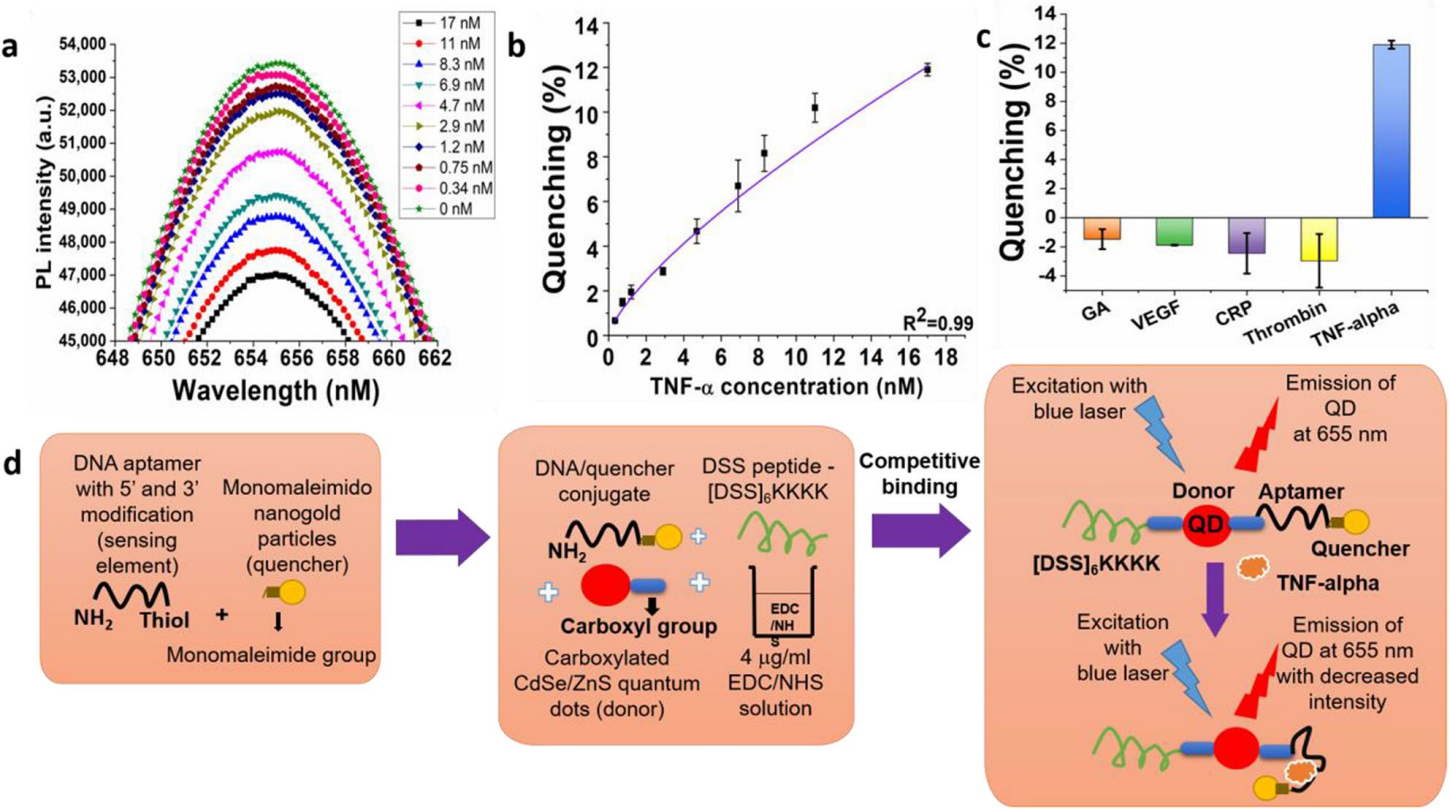
Decrease in photoluminescence intensity with increase in TNF-α in DSS-molecular beacon conjugate (**a**). The quenching effect has been shown with respect to the increase in the target protein concentration in the sensor (**b**). Specificity of sensor to TNF-α with respect to control proteins (**c**). Synthesis strategy and possible structure of DSS conjugated molecular beacons along with their detection strategy (**d**).

The sensor is composed of a semiconductor quantum dot, which is an excellent donor for FRET applications. The primary sensing element, which binds to the target molecule is a nucleic acid based aptamer. Gold nanoparticles have been employed as quenchers here due to their wide absorption spectrum. The DSS peptide denotes the sequence DSSDSSDSSDSSDSSDSSKKKK, which has been shown previously to induce endocytosis^26^ and which we have used here to cause endocytosis of the molecular beacon into the cell. The synthesis procedure and the possible structure of the DSS-molecular beacon conjugate has been summarized in Fig. 2d. As can be observed in Fig. 2a, the photoluminescence intensity exhibited by the DSS-molecular beacon conjugate decreases with an increase in TNF-α concentration. The molecular beacon is composed of a DSS conjugated quantum dot linked to a gold nanoparticle via a short DNA aptamer, which binds to TNF-α. The DNA aptamer changes its conformation once the target analyte is added to the sensor solution; this results in bringing the quantum dot (donor) closer to the gold nanoparticle (quencher). This causes an intersystem energy transfer from the donor to the quencher, known as FRET. Therefore, an increase in quenching is observed with an increase in target protein concentration (Fig. 2b).

The DSS conjugated molecular beacon was observed to be highly specific to TNF-α when compared to control proteins. As shown in Fig. 2c, the quenching exhibited by the sensor towards TNF-α was higher than that exhibited by the control proteins. Control proteins such as GA and CRP were tested at a concentration of 1 μM while Thrombin and VEGF were tested at 65 nM concentration. The quenching exhibited by the sensor towards these control proteins was compared to that exhibited by 17 nM TNF-α. Based on the observations in Fig. 2c, the control proteins demonstrated an increase in the PL intensity while in case of the target protein, and there was a significant decrease in PL intensity. Since the quenching behavior of the sensor was significantly different for TNF-α and the control proteins, it was concluded that there would be no cross reactivity at other concentrations of the control proteins. These results have been compared with the control testing data reported by Ghosh et al.^1^, where they tested the specificity of the TNF-α detecting molecular beacon. This molecular beacon did not have the DSS peptide attached to it. We, Ghosh et al.^1^, found and published that the beacon was highly specific to the target protein when compared to control proteins such as C-reactive protein (CRP), albumin, transferrin and thrombin. This establishes that the presence of the DSS peptide does not have an effect on the detection mechanism of the molecular beacon.

### Testing DSS peptide conjugated molecular beacons with cells

#### For GA detecting molecular beacons

**Figure 3:**
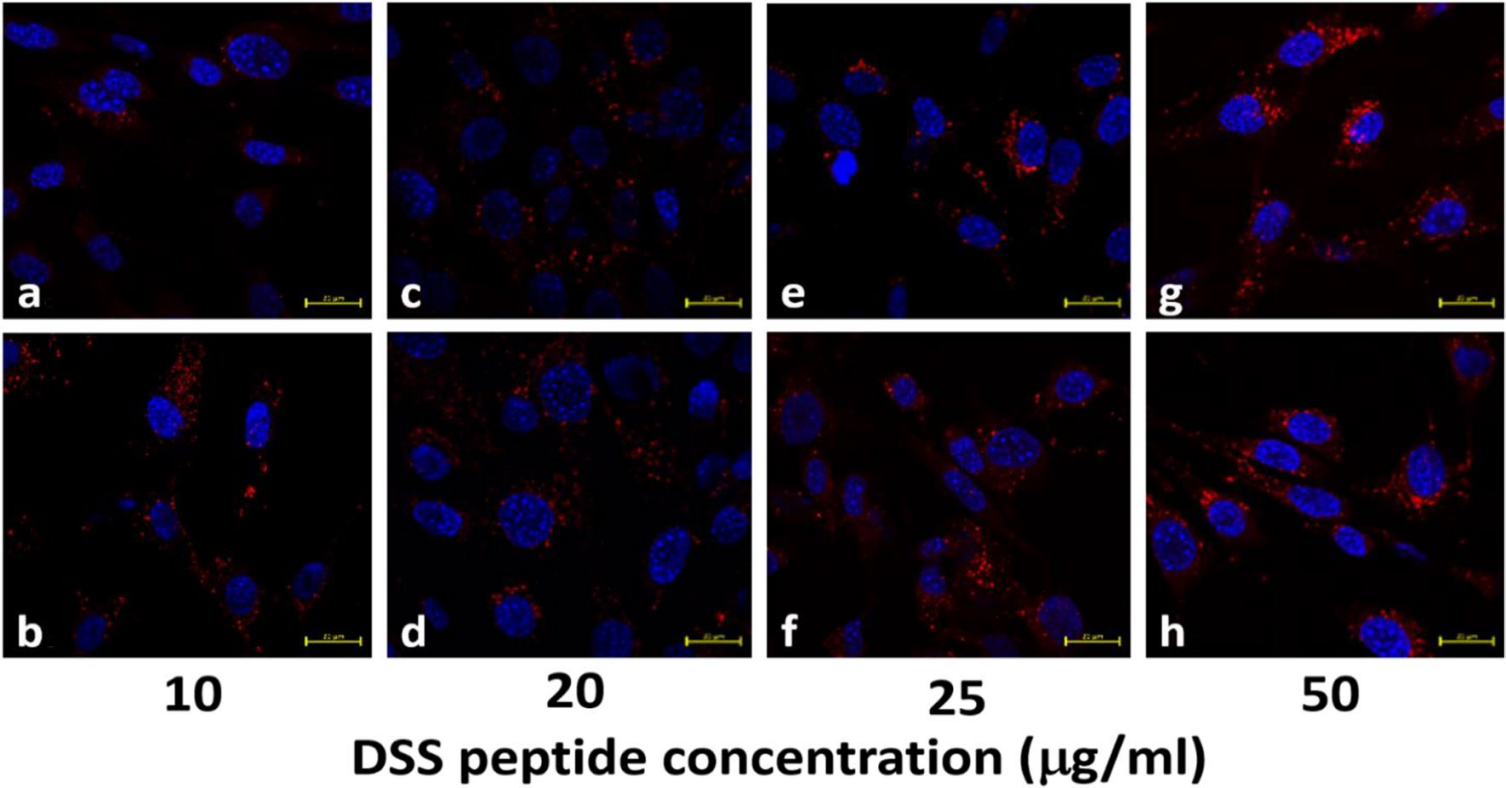
Interaction of DSS peptide conjugated GA detecting molecular beacons with MC3T3 cells at different concentrations of the peptide used during the cell culture testing. The concentration of the 655 nm quantum dots (red) around the cell nucleus (blue) is compared in the presence of (**a**,**b**) 10 μg/ml, (**c**,**d**) 20 μg/ml, (**e**,**f**) 25 μg/ml, and (**g**,**h**) 50 μg/ml concentration of the DSS peptide.

The purpose of this test was to determine whether there was a successful conjugation of the DSS peptide to the GA detecting aptasensors. Ravindran et al. previously showed that the DSS peptide facilitates cellular uptake^26^. To reduce peptide wastage, three different concentrations of the peptide was initially tested to determine the optimal concentration at which future experiments would be conducted. The working concentrations of the DSS peptide used during the cell culture process is shown in Supplementary Table S2. As shown in Fig. 3a–h, the quantum dots in the molecular beacon was observed to be surrounding the nucleus for all the four working concentrations of the peptide (10 μg/ml, 20 μg/ml, 25 μg/ml and 50 μg/ml). However, based on the concentration of the quantum dots around the cell nucleus in Fig. 3a–h, it was determined that the best concentration of the DSS peptide for future endocytosis experiments was 50 μg/ml. Hence, the DSS stock concentration of 10 mg/ml was used in future synthesis protocols of the DSS peptide conjugated aptamer sensor.

#### For TNF-α detecting molecular beacons

**Figure 4:**
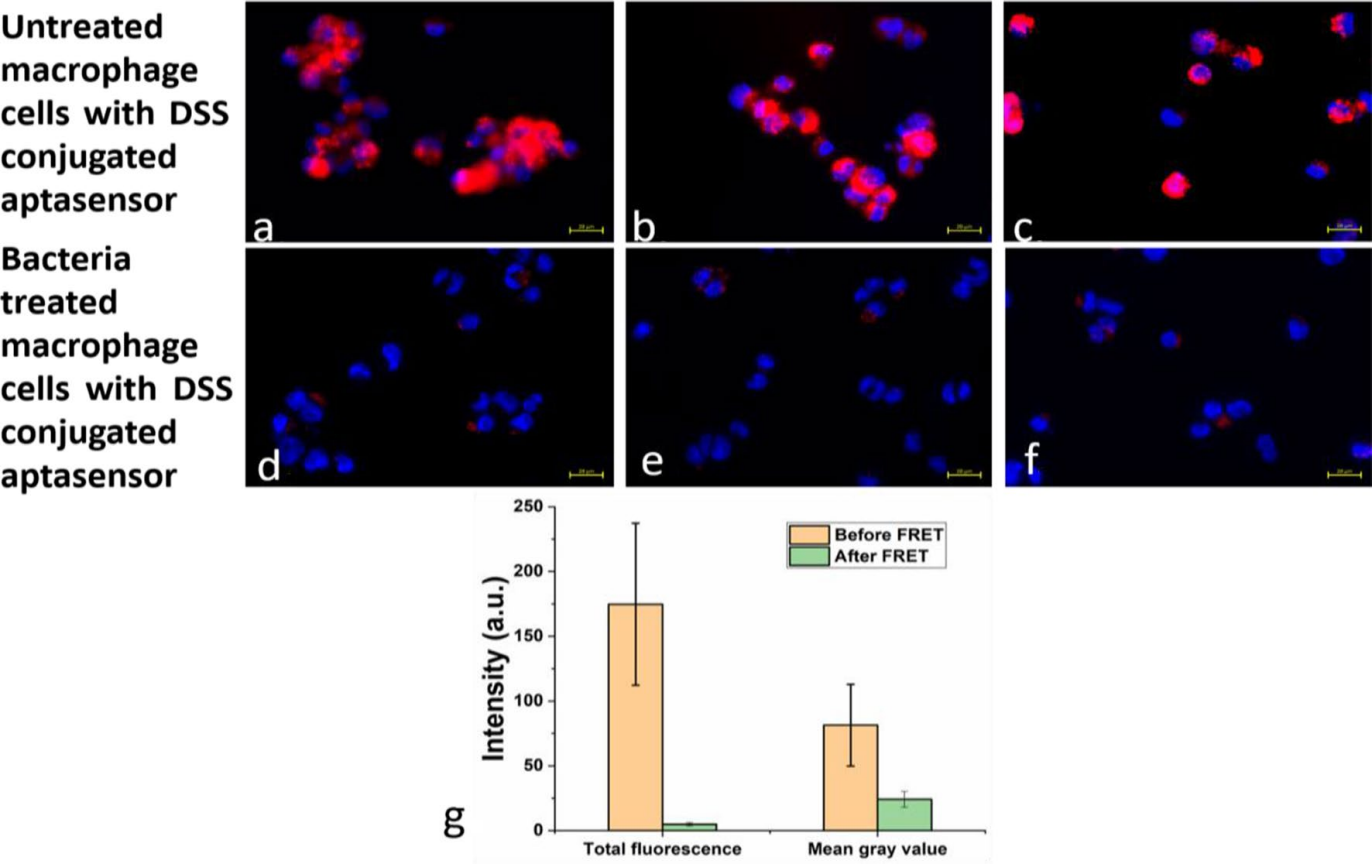
The phenomenon of FRET has been illustrated here. A decrease in fluorescence intensity is observed when the sensor interacts with the bacterial lipopolysaccharide treated macrophage cells in (**d**–**f**) compared to the non-infected macrophage cells in (**a**–**c**). Comparison of fluorescence intensities and mean gray value intensities before and after FRET in the RAW 264.7 cells (**g**).

Figure 4a–c shows macrophage cells (RAW 264.7) in the presence of the DSS peptide conjugated molecular beacon. This molecular beacon incorporated a DNA aptamer which specifically detects TNF-α. In Fig. 4d–f, the macrophage cells were treated with bacterial lipopolysaccharide, which induced an infection and hence, resulted in the intracellular production of the pro-inflammatory cytokine. As can be observed in Fig. 4d–f, there was a decrease in the fluorescence intensity emitted by the quantum dots when compared to the emission observed on Fig. 4a–c. The total fluorescence intensities in Fig. 4a–f has been plotted in Fig. 4g. The fluorescence emission intensities from the quantum dot for Fig. 4a–c remains approximately 175 units (Fig. 4g), indicating a high quantum dot emission without the presence of a high concentration of TNF-α. A huge decrease in the fluorescence intensity is observed once the DSS peptide conjugated molecular beacon interacts with the infected macrophage cells. A similar trend is observed with the mean gray values (Fig. 4g). The total fluorescence intensities and the mean gray values obtained from Image J and also utilized for plotting Fig. 4g have been summarized in Supplementary Table S3. The reduction in fluorescence intensity indicated the phenomena of quenching taking place inside the cells in the presence of a higher concentration of TNF-α. Therefore, it was concluded that the DNA aptamer based molecular beacons were undergoing FRET and were successfully detecting TNF-α in the presence of bacterial infection. This observation was attributed to the detection strategy of the molecular beacon shown in Fig. 2d. Hence, the reduction in the fluorescence intensity shown in Fig. 4a–f is in agreement to the calibration photoluminescence curve and quenching curve shown in Fig. 2a,b.

**Figure 5:**
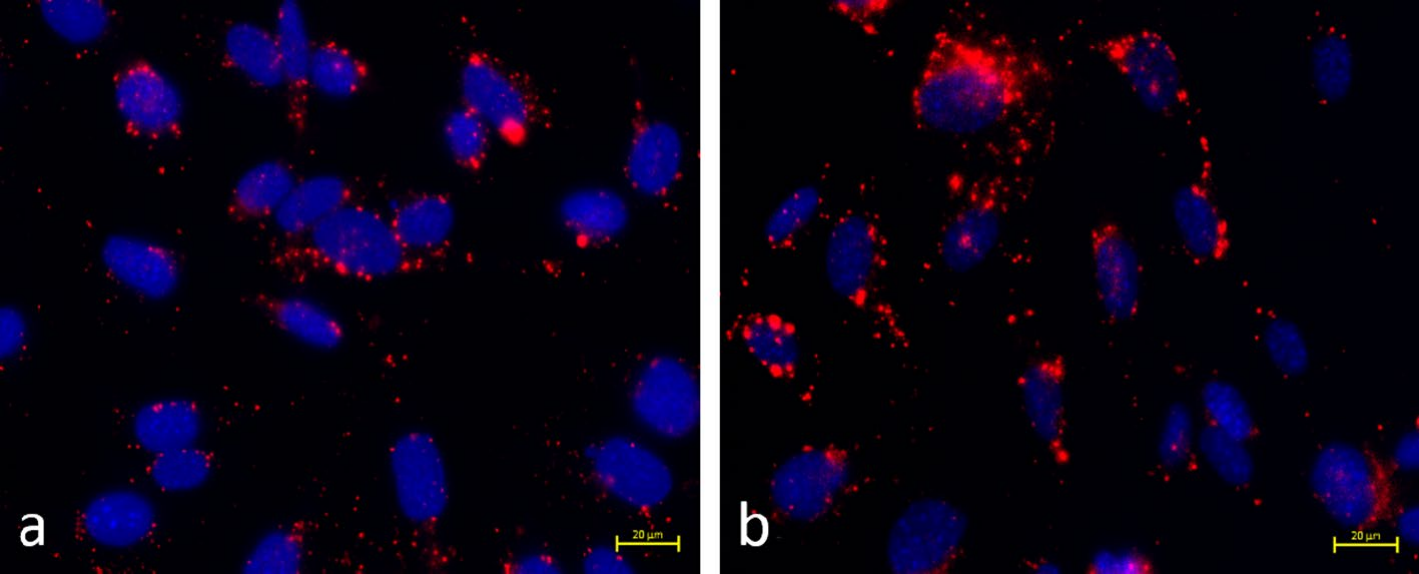
The specificity of the aptasensors is proven when they do not show any quenching in osteoblast cells, which do not produce any TNF-α. (**a**,**b**) Different regions in the cells having high quantum dot emission.

The specificity of the DSS conjugated molecular beacons were determined by testing the sensors with the MC3T3 cells. These cells do not produce TNF-α. As can be observed in Fig. 5a,b, there is no change in the emission of the quantum dots indicating the absence of any quenching phenomena. Hence, FRET was observed only in the presence of inflammation, which induces increased production of intracellular TNF-α. Ghosh et al. have already showed that the original molecular beacon was specific to TNF-α when compared to other biomarker proteins like CRP, Transferrin etc. except for demonstrating a significant cross reactivity towards thrombin^1^. However, the results shown in Figs. 4 and 5 indicates that the peptide conjugated sensor can successfully detect TNF-α even in the presence of thrombin in the cellular environment. This showed that the DSS conjugated molecular beacon is highly specific towards detecting TNF-α in an inflammatory environment.

The sensor reported in this paper consists of a DSS peptide and molecular beacon specific for the detection of TNF-α. The DSS peptide is a cell penetrating peptide capable of dragging a cargo inside the cell towards the nucleus. The molecular beacon consists of a DNA aptamer as the primary sensing element and operates on the principle of FRET. This peptide-molecular beacon nanoconstruct can successfully detect TNF-α inside the macrophage cells. The novelty of this sensor lies in its design. The DSS peptide can be conjugated to various kinds of biomolecules like drugs. Hence, the nanoconstruct reported in this paper can be potentially used in future drug delivery applications. This will also open up new avenues in the field of theranostics. Besides, it also enjoys the flexibility of being able to detect other biomarker proteins by just replacing the sensing element (DNA aptamer).

## Methods

### Aptamer structure and solution preparation

The GA binding ssDNA aptamer and the TNF-α binding ssDNA aptamer used in this study was purchased from Biosearch Technologies (Petaluma, CA, USA). The aptamers used in this study have been chosen based on literature review and previous studies conducted on them. The GA binding aptamer consists of 23 bases and has been modified on both ends with an amine group on the 5′ and a thiol group on the 3′ (5′Amino C6/TGCGGTTGTAGTACTCGTGGCCG/Thiol C6 SS 3′)^2^. The aptamer was dissolved into tris ethylenediamine tetraethyl acetate (EDTA) buffer to obtain 100 µM aptamer solution. This step was conducted in order to prevent cation induced degradation of DNA bases.

The TNF-α binding aptamer consists of 25 bases and has been modified on both ends with an amine group on the 5′ and a thiol group on the 3′ (5′AminoC6/TGGTGGATGGCGCAGTCGGCGACAA/Thiol C6 SS 3′)^1^. To obtain a 100 µM aptamer solution, the aptamer was dissolved into 887 μl of EDTA buffer.

### Materials used for synthesis protocols

#### Synthesis of DSS peptide conjugated molecular beacon using GA binding aptamer

The synthesis protocol of the DSS conjugated molecular beacon with the GA binding aptamer is based on the protocol reported by Ghosh et al.^2^. Briefly, 9 μl of TCEP was added to 20 μl of the 100 μM GA aptamer and the mixture was allowed to incubate for 30 min at room temperature. This step facilitated the reduction of the dithiol groups in the aptamer. 100 μl of gold nanoparticle (diameter: 1.4 nm; Nanoprobes, USA)^27^ solution was synthesized by adding the same volume of de-ionized water to one vial of gold nanoparticles. This was further added to the aptamer-TCEP mixture in order to achieve a 3:1 ratio between the quencher and the aptamer. The resulting solution was then incubated for 2 h at room temperature. Subsequently, it was centrifuged twice at 5,000 rpm for 15 min each using a 3 k MWCO filter in order to remove the excess unbound gold nanoparticles from the solution. After each centrifugation, 50 μl of de-ionized water was used to wash the supernatant. The centrifuge used for all centrifugation steps was the Fisher Scientific Accuspin micro (Fisher Scientific, USA). A 100 μl QD solution was synthesized by mixing 87 μl of 10 mM borate buffer (pH 7.4) and 13 μl of carboxylated CdSe/ZnS QD (0.1 nmoles). The carboxylated CdSe/ZnS QDs (diameter: 20 nm) used in this protocol as well the other protocols, stated in the paper were obtained from Thermo Fisher Scientific (Qdot 655 ITK—8 μM solution—2 nmoles in 50 mM borate buffer)^28^ and the datasheets show a sharp emission spectrum centered at 655 nm with a half-width of 35 nm. Such a narrow emission spectrum is a general feature of high quality QDs since the dominant quantum mechanical emission probability is from the bottom of the conduction band to the top of the valence band. The gold nanoparticles used in these experiments have a wide absorption spectrum^27^ with the ability to absorb light between 300 and 800 nm. Therefore, they are effective at quenching fluorescence at 655 nm, which is the peak emission wavelength of the QD. Four different amounts of DSS peptide was added during four different synthesis experiments of the peptide conjugated sensor. The concentrations of the peptide are summarized in Table 1. EDC/NHS coupling chemistry was used to bind the QDs to the DNA aptamer as well as to bind the DSS peptide to the QD. 100 μl of the QD solution and 230 μl of DSS peptide solution (with the corresponding concentration mentioned in Table 1) was added to the filtered GA aptamer/gold nanoparticle solution in the presence of 30 μl of 4 μg/μl EDC/Sulpho NHS solution. The resulting solution was then allowed to shake for 2 h at room temperature. Subsequently, the samples were centrifuged five times at 7,000 rpm for 5 min each using a 100 k MWCO filter in 50 mM borate buffer (pH 8.3). The supernatant was washed with 50 μl of the 50 mM borate buffer (pH 8.3) after each centrifugation. This step allowed the unbound aptamers and excess EDC to get eliminated from the sensor solution.

**Table 1:** Summary of three different concentrations of DSS peptide added during synthesis of the sensor–peptide conjugate.

Serial number	Amount of DSS peptide (mg)	Volume of water (μl)	Concentration of peptide added to the sensor (mg/ml)
1	0.46	230	2
2	0.92	230	4
3	1.15	230	5
4	2.3	230	10

#### Synthesis of DSS peptide conjugated molecular beacon using TNF-α binding aptamer

The synthesis protocol of the DSS conjugated molecular beacon with the TNF-α binding aptamer is based on the protocol reported by Ghosh et al.^1^. Briefly, 9 μl of TCEP was added to 20 μl of the 100 μM TNF-α aptamer and the mixture was allowed to incubate for 30 min at room temperature. 100 μl of gold nanoparticle (diameter: 1.4 nm; Nanoprobes, USA) solution was synthesized by adding the same volume of de-ionized water to one vial of gold nanoparticles. This was further added to the aptamer-TCEP mixture in order to achieve a 3:1 ratio between the quencher and the DNA aptamer. The resulting solution was then incubated for 2 h at room temperature. Subsequently, it was centrifuged twice at 5,000 rpm for 15 min each using a 3 k MWCO filter in order to remove the excess unbound gold nanoparticles from the solution. After each centrifugation, 50 μl of de-ionized water was used to wash the supernatant. The centrifuge used for all centrifugation steps was the Fisher Scientific Accuspin micro (Fisher Scientific, USA). A 100 μl QD (diameter: 20 nm; Thermo Fisher Scientific, USA) solution was synthesized by mixing 87 μl of 10 mM borate buffer (pH 7.4) and 13 μl of carboxylated CdSe/ZnS QD. 2.3 mg of the DSS peptide was added to 230 μl of de-ionized water in order to make a 10 mg/ml peptide solution. EDC/NHS coupling chemistry was used to bind the QDs to the DNA aptamer as well as to bind the DSS peptide to the QD. 100 μl of the QD solution and 230 μl of the DSS peptide was added to the filtered TNF-α aptamer/gold nanoparticle solution in the presence of 30 μl of 4 μg/μl EDC/Sulpho NHS solution. The resulting solution was then allowed to shake for 2 h at room temperature. Subsequently, the samples were centrifuged five times at 7,000 rpm for 5 min each using a 100 k MWCO filter in 50 mM borate buffer (pH 8.3). The supernatant was washed with 50 μl of the 50 mM borate buffer (pH 8.3) after each centrifugation. This step allowed the unbound aptamers and excess EDC to get eliminated from the sensor solution.

### Characterization of DSS peptide structure

The DSS peptide was characterized using Raman spectroscopy. 5 μl of the DSS peptide was allowed to dry on a stainless steel Raman substrate. Raman measurements were performed with a Renishaw inVia Reflex Raman spectrometer using 532 nm HeNe laser excitation (17.5 mW laser power output) with 1% laser power, long working distance 50 × objective (8.2 mm WD), 10 s exposure time and 3 accumulations for each run.

### Testing DSS peptide conjugated molecular beacons with PBS based TNF-α samples

#### Sensitivity characterization

The TNF-α solutions were prepared by diluting the 10 μg/ml in PBS. 5 μl of these solutions were added to the 750 μl sensor solution. The sensor–target mixture was then allowed to incubate for an hour. The photoluminescence intensities were obtained using a USB4000 Ocean Optics (Dunedin, FL, USA) spectrophotometer with a continuous 375 nm LED excitation.

#### Specificity characterization

The DSS conjugated molecular beacons were tested using control proteins such as GA, vascular endothelial growth factor (VEGF), C-reactive protein (CRP), and Thrombin. The control protein solutions were prepared in PBS. All the proteins were kept at a concentration of 65 nM or above except TNF-α, which was kept at 17 nM. 5 μl of these solutions were added to the 750 μl sensor solution. The sensor–protein mixture were then allowed to incubate for an hour. The photoluminescence intensities were obtained using a USB4000 Ocean Optics (Dunedin, FL, USA) spectrophotometer with a continuous 375 nm LED excitation.

### Testing DSS peptide conjugated molecular beacons with cells

#### For GA detecting molecular beacons

Mouse pre-osteocyte cells (MC3T3 E1) were cultured in α-MEM with 10% FBS and 1% Antibiotic–Antimycotic (100×, Life technologies) at 37 °C in a humidified incubator with 5% CO_2_. The 250,000 cells were seeded on a ϕ 25 mm cover glass in a well of 6 well culture plate. The next day the DSS conjugated GA detecting aptasensors were added to interact with the cells.

#### For TNF-α detecting molecular beacons

Mouse monocyte/macrophage cells (RAW264.7) were a kind gift from Dr. Afsar Naqvi (University of Illinois at Chicago) and were cultured in DMEM with 10% FBS and 1% Antibiotic–Antimycotic at 37 °C in a humidified incubator with 5% CO_2_. The 100,000 cells were seeded on a ϕ 12 mm cover glass in a well of 24 well culture plate. The cells were then stimulated/differentiated by lipopolysaccharides (100 ng/ml in RPMI media—Escherichia coli O26:B6–BSL 2, Invitrogen) in the same culture media for 4 h. Subsequently, the DSS conjugated TNF-α detecting aptasensors were added to interact with the cells.

After addition of QDs, the reactions were stopped briefly rinsed with PBS twice, subsequently the cells were fixed with 10% formalin (PBS neutralized) at 37 °C for 1 h. After washing with PBS for 3 times, the cover glass was mounted on a glass slide with mounting agent with DAPI (VectorLab). The QD fluorescence signals were observed with a Zeiss LSM 710 Confocal Microscope in Research Resources Center of University of Illinois at Chicago or with a Zeiss Observer D1 Microscope.

The QD fluorescence images were processed using MATLAB R2019a to obtain the filtered images of the quantum dot emissions. These filtered were further processed using Image J software to obtain the total fluorescence intensities and mean gray values.

## Supplementary information

Supplementary file1

## Data Availability

All data generated or analyzed during this study are included in this article (and its Supplementary Information files).
