# Supplementary material for: A study on the response of FRET based DNA aptasensors in intracellular environment
Source: Sci Rep. 2020 Aug 6;10:13250. doi: 10.1038/s41598-020-70261-1 (PMC7413375; doi:10.1038/s41598-020-70261-1)
Supplement: Supplementary file 1 — Supplementary file1 [file 41598_2020_70261_MOESM1_ESM.pdf]

A study on the response of FRET based DNA aptasensors in intracellular environment

Shreya Ghosh[1*], Yinghua Chen[2], Jesvin Sebastian[1], Anne George[2], Mitra Dutta[3, 4], Michael A. Stroscio[1, 3, 4]

[1]Department of Bioengineering, University of Illinois at Chicago, 851 South Morgan Street (SEO 218), Chicago, Illinois, 60607, USA.

[2]Department of Oral Biology, University of Illinois at Chicago, 801 South Paulina Street, Chicago, Illinois, 60612, USA.

[3]Department of Electrical and Computer Engineering, University of Illinois at Chicago, 851 South Morgan street, M/C 154, Chicago, Illinois, 60607, USA.

[4]Department of Physics, University of Illinois at Chicago, 845 W. Taylor St., M/C 273, Chicago, Illinois, 60607, USA.

SUPPLEMENTARY INFORMATION

Supplementary Table S1. Peaks and their possible assignments in the Raman spectra of the DSS peptide.

**Wavenumber (cm^{-1})**	**Possible peak assignments**	**References**
731	COO^- bending in aspartic acid	[20]
838	C-C and C-N stretching in aspartic acid	[21]
918	C_α-C_β stretching in serine	[16,22]
1022	Possible β - sheet structure assignment	[23]
1054	C_α–C stretching / CH_2 rocking (out of plane) in serine	[22,24]
1155	CH_2 twisting in serine	[22]
1232	Amide 3 region (C-N stretching and N-H bending)	[15]
1296	C-H bending in serine	[24]
1564	Amide 2 region/ COO^- stretching in aspartic acid/lysine	[15,16]
1614	NtH_3^+ asymmetric bending in lysine	[16]
1671	Amide 1 region (antiparallel beta sheet structure)	[15]
2941	C-H stretching in lysine	[25]

Supplementary Table S2. Final concentrations of DSS peptide used during cell culture.

Serial number	Stock Concentration (mg/ml)	Dilution factor	Working concentration (mg/ml)	Working concentration (µg/ml)
1.	2	200	0.0100	10
2.	4	200	0.0200	20
3.	5	200	0.0250	25
4.	10	200	0.0500	50

MATLAB code for RGB manipulation and Image filtering:

```
%defines all filename
filename = 'QD fig 3a QD control tnf dss-3_c1+2.tif';
filename2 = 'QD fig 3b QD control tnf dss-2_c1+2.tif';
filename3 = 'QD fig 3c QD control tnf dss_c1+2.tif';
filename4 = 'QD fig 3d QD tnf cells DSS-3_c1+2.tif';
filename5 = 'QD fig 3e QD tnf cells DSS-2_c1+2.tif';
filename6 = 'QD fig 3f QD tnf dss.-3czi_c1+2.tif';

[original, filtered] = photoFilter(filename, "red");% calls function for maintaining red RGB value
imshow(filtered); % displays the new filtered image
title("red") % gives the image a title

function [original, filtered] = photoFilter(filename, opt)
original = imread(filename);% causes image to become an 3D array
filtered = original; %filtered image is set as original image
filtered(:, :, 2) = 0;% sets green layer to 0
filtered(:, :, 3) = 0;% sets blue layer to 0
end
```

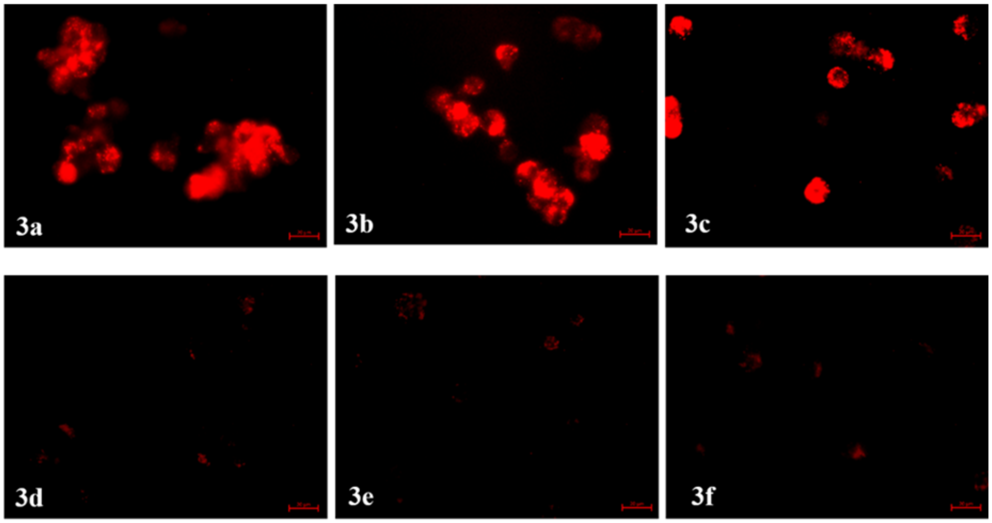

Supplementary Figure S1. Filtered images obtained using MATLAB code. These figures (a-f) isolate the quantum dot emissions, illustrated in Fig. 4(a-f) respectively. These images have been further analyzed using Image J to generate Supplementary Table S1.

Supplementary Table S3. Summary of the total fluorescence and mean gray values obtained from the quantum dot emission in Supplementary Fig. S1 (a-f), which has been extracted from Fig. 4(a-f) respectively. The data establishes the phenomenon of quenching during FRET, caused by the DSS peptide conjugated molecular beacon inside the cells. Avg. stands for average while Std. stands for standard deviation

**Before FRET**					
Figure	3a	3b	3c	Avg.	Std.
Total fluorescence intensity	240.322	167.855	116	174.726	62.445
Mean gray value	55.046	72.935	116.374	81.451	31.538
**After FRET**					
Figure	3d	3e	3f	Avg.	Std.
Total fluorescence intensity	3.535	5.453	5.872	4.953	1.246
Mean gray value	21.671	19.745	31.098	24.171	6.075
